# Epigenetics in cancer: from mechanisms to precision medicine

**DOI:** 10.1002/1878-0261.70340

**Published:** 2026-10-06

**Authors:** Sung Hee Baek

**Affiliations:** ^1^ School of Biological Sciences Seoul National University Republic of Korea

**Keywords:** ADP‐ribosylation, arginine methylation, cancer, chromatin regulation, epigenetics, spatial epigenomics, tumor plasticity, whole‐genome doubling

## Abstract

Epigenetics encompasses heritable yet reversible changes in gene expression that occur without alterations to the underlying DNA sequence. Through coordinated regulation of DNA methylation, histone modifications, chromatin remodeling, and higher‐order chromatin organization, epigenetic mechanisms shape chromatin accessibility and transcriptional programs that govern cellular identity, plasticity, and tissue homeostasis. In cancer, disruption of the epigenetic landscape has emerged as a hallmark of tumor initiation, progression, metastasis, immune evasion, and therapeutic resistance. Unlike genetic alterations, epigenetic abnormalities are potentially reversible, making them attractive therapeutic targets. Recent advances in next‐generation sequencing, single‐cell and spatial multi‐omics, chromatin profiling technologies, and computational biology have transformed our understanding of cancer epigenomes while accelerating the development of epigenetic therapies targeting diverse chromatin regulatory pathways. This Thematic Issue on Epigenetics in Cancer brings together leading experts to review recent conceptual and technological advances that are reshaping the field. Covering topics ranging from spatial and single‐cell epigenomics to chromatin regulation, tumor plasticity, and emerging therapeutic strategies, the collection highlights how fundamental discoveries are driving translational innovation and precision oncology. We hope these articles will provide a valuable resource for researchers and clinicians while inspiring future advances in cancer epigenetics and epigenetic therapeutics.

## Graphical abstract

Reversible epigenetic programs, including DNA methylation, chromatin modifications, and chromatin architecture, shape dynamic tumor states. Spatial and single‐cell profiling identify tumor plasticity, evolution, and immune evasion, enabling biomarker‐guided precision medicine and rational epigenetic combinations to expose therapeutic vulnerabilities. This article provides an overview on the Thematic Issue on Epigenetics in Cancer that covers some of the most exciting developments in the field. The image was generated with the assistance of AI.

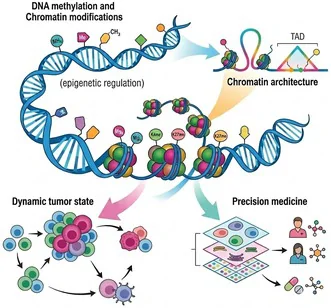

**Abbreviations**
ATAC‐seq: assay for transposase‐accessible chromatin using sequencing
CUT&RUN: cleavage under targets and release using nuclease
CUT&Tag: cleavage under targets and tagmentation
DNA: deoxyribonucleic acid
EMT: epithelial–mesenchymal transition
WGD: whole‐genome doubling

## 1 Overview of this thematic issue

The eight reviews featured in this Thematic Issue on Epigenetics in Cancer reflect the extraordinary progress of the field, spanning fundamental discoveries in chromatin biology, emerging mechanistic insights, and their translation into precision oncology. Together, they capture several emerging themes that are reshaping the field, including advances in spatial and single‐cell epigenomics, epigenetic regulation of tumor evolution and plasticity, novel chromatin‐associated regulatory mechanisms, and the development of therapeutic strategies that exploit epigenetic vulnerabilities (Fig. 1).

**Fig. 1:**
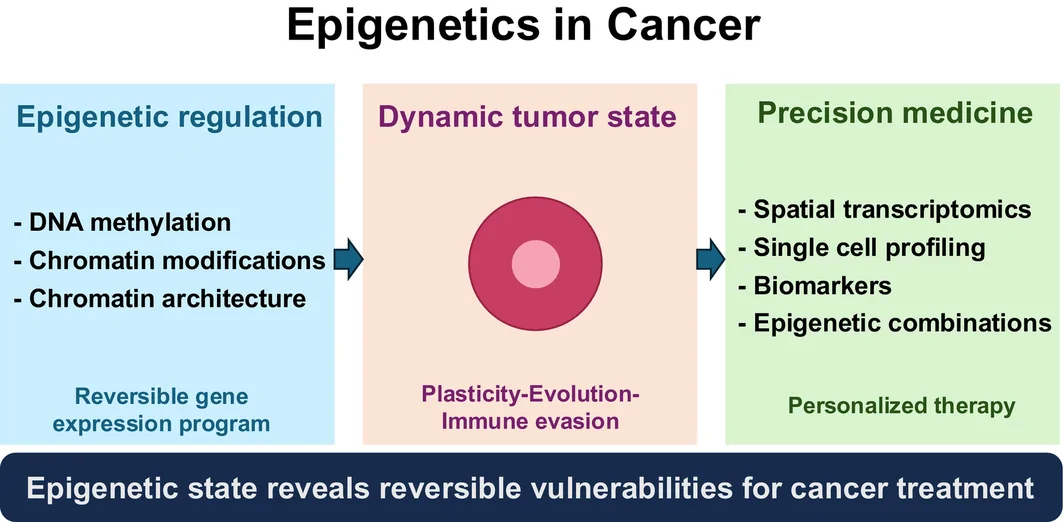
Epigenetics in cancer. The schematic summarizes how reversible epigenetic programs, including DNA methylation, chromatin modifications, and chromatin architecture, shape dynamic tumor states. Spatial and single‐cell profiling identify tumor plasticity, evolution, and immune evasion, enabling biomarker‐guided precision medicine and rational epigenetic combinations to expose therapeutic vulnerabilities.

## 2 Emerging technologies

A major focus of this issue is the rapid emergence of technologies that enable epigenetic regulation to be investigated with unprecedented spatial and cellular resolution. Manel Esteller reviews the rapidly advancing field of spatial epigenomics, highlighting innovative approaches such as spatial ATAC‐seq, spatial CUT&Tag, emerging spatial CUT&RUN approaches, spatial DNA methylation profiling, and multimodal platforms that preserve tissue architecture while interrogating chromatin regulation. Complementing these spatial approaches, Ik Soo Kim discusses recent advances in single‐cell DNA methylation profiling, comparing emerging experimental and computational strategies for reconstructing cellular lineages, identifying therapy‐resistant subpopulations, and developing DNA methylation‐based biomarkers. By profiling the epigenome of individual cells, single‐cell DNA methylation analysis enables the discovery of novel aberrant patterns, the precise reconstruction of cellular lineages, and the characterization of specific populations such as cancer stem cells or drug‐resistant clones that possess distinct methylome signatures. Together with the review by Daechan Park, which examines how single‐cell multi‐omics has transformed our understanding of epigenetic heterogeneity and therapy‐induced cellular plasticity, these articles illustrate how next‐generation epigenomic technologies are redefining our ability to dissect tumor ecosystems, lineage evolution, and dynamic cell‐state transitions at unprecedented resolution. It will be interesting to speculate how integrative multi‐layer analyses enable comprehensive characterization of tumor‐state evolution, providing a conceptual framework for precision oncology strategies aimed at overcoming resistance [1, 2, 3].

## 3 Epigenetic mechanisms

This issue also highlights several fundamental chromatin‐associated regulatory mechanisms that are increasingly recognized as central drivers of malignant gene expression programs. Lee Kraus provides an overview of ADP‐ribosylation as an emerging epigenetic regulator that extends well beyond its classical role in DNA damage responses to influence chromatin organization, transcriptional regulation, and epigenome remodeling. The authors summarize emerging insights into nuclear ADP‐ribosylation, with a focus on mono ADP‐ribosyl transferases‐mediated regulation of histones and chromatin enzymes, and discuss how this regulatory layer expands current models of epigenomic control in physiology and how its alterations drive oncogenesis, offering novel, non‐synthetically lethal avenues for targeted cancer therapy. Complementing this perspective, Ji Min Lee introduces the concept of arginine methylation as a “regulatory ratchet,” describing how substrate‐specific methylation progressively stabilizes malignant cellular states and reinforces oncogenic transcriptional programs. The authors propose a regulatory ratchet framework in which protein arginine methyltransferase‐dependent methylation can reinforce cellular states through cumulative and network‐level effects rather than acting as a binary switch. Together, these reviews emphasize the growing complexity of chromatin regulation and reveal previously underappreciated mechanisms that may represent attractive therapeutic targets [4, 5].

## 4 Tumor evolution and microenvironment

Beyond molecular mechanisms, several reviews address how epigenetic regulation shapes tumor evolution and interactions with the surrounding microenvironment. Jinhyuk Bhin discusses whole‐genome doubling as a pivotal evolutionary event that simultaneously promotes chromosomal instability and creates exploitable therapeutic vulnerabilities, providing a conceptual framework for translating whole‐genome doubling biology into precision oncology. This review provides how whole‐genome doubling arises and drives tumor evolution, its prevalence and prognostic impact across cancer types, and the emerging therapeutic vulnerabilities that whole‐genome doubling‐positive tumors expose. Mi‐Young Kim explores the reciprocal epigenetic regulation governing bidirectional communication between cancer cells and the tumor microenvironment, highlighting how chromatin‐mediated interactions influence tumor progression and therapeutic response. The authors discuss a variety of epigenetic mechanisms controlling cancer cell‐intrinsic states, cancer cell–tumor microenvironment crosstalk, current epigenetic therapies, and future research directions that may lead to the discovery of new biomarkers and the development of effective epigenetic anticancer therapeutics. These concepts are closely linked to the review by Daechan Park, which illustrates how epigenetic plasticity enables tumor cells to adapt under therapeutic pressure, giving rise to drug‐tolerant persister cells, epithelial–mesenchymal transition, senescence‐like states, and immune‐evasive phenotypes [3, 6, 7].

## 5 Translation into therapeutics

Finally, this collection underscores the expanding opportunities to translate epigenetic knowledge into innovative therapeutic strategies. In an intriguing cross‐disciplinary perspective, Xiaodong Cheng demonstrates how decades of work on fetal hemoglobin regulation have established clinically successful approaches for manipulating transcription factors and chromatin regulators. By drawing conceptual parallels between hemoglobinopathies and cancer, this review illustrates how shared regulatory factors may inspire novel approaches for targeting transcriptional and epigenetic dependencies in malignant cells. Together with the advances in epigenomic profiling and mechanistic insights presented throughout this issue, these studies highlight the increasing convergence of fundamental epigenetic discovery and precision cancer medicine [8].

## 6 Concluding remarks

Collectively, the reviews assembled in this Thematic Issue underscore a defining transition in cancer epigenetics: from describing static molecular alterations to understanding—and ultimately intervening in—the dynamic regulation of tumor cell states. Advances in spatial, single‐cell, and multi‐omic technologies now enable the epigenetic landscape to be examined within its native tissue context and at a resolution capable of revealing rare cell populations, lineage relationships, and therapy‐induced state transitions. These approaches offer an increasingly powerful framework for deciphering how intratumoral heterogeneity, cellular plasticity, and microenvironmental interactions shape tumor evolution and treatment failure.

At the same time, emerging insights into chromatin‐associated mechanisms, including ADP‐ribosylation and arginine methylation, expand the regulatory repertoire through which malignant transcriptional programs are established and maintained. Together with studies of whole‐genome doubling, tumor–microenvironment crosstalk, and drug‐tolerant cellular states, these perspectives emphasize that cancer is not defined solely by genetic lesions, but also by reversible and adaptive epigenetic programs. Such programs may create vulnerabilities that are context‐dependent, transient, and therefore potentially actionable when identified with sufficient precision.

A central challenge ahead will be to translate this growing mechanistic and technological knowledge into clinically meaningful strategies. This will require longitudinal and integrative analyses that connect epigenetic states with genetic background, cellular phenotype, immune context, therapeutic exposure, and clinical outcome. It will also require the development of robust biomarkers to identify the patients, tumor states, and treatment windows most likely to benefit from epigenetic intervention. Rather than viewing epigenetic therapies simply as broadly acting chromatin‐modifying agents, the field is moving toward rational combinations designed to reprogram tumor cell states, prevent adaptive resistance, enhance antitumor immunity, or expose lineage‐specific dependencies.

The lessons from successful manipulation of transcriptional and chromatin regulators in non‐malignant disease further reinforce the therapeutic potential of this approach. As fundamental discoveries increasingly converge with precision oncology, epigenetic research is poised to deliver not only new targets but also new principles for treating cancer as an evolving ecosystem of cellular states. We hope that this collection will stimulate continued interdisciplinary efforts to define these principles and translate them into more durable, personalized, and effective cancer therapies.

## Conflict of interest

The author declares no conflict of interest.

## Author contributions

SHB wrote the manuscript.
